# Evaluating Hepatobiliary Transport with ^18^F-Labeled Bile Acids: The Effect of Radiolabel Position and Bile Acid Structure on Radiosynthesis and* In Vitro* and* In Vivo* Performance

**DOI:** 10.1155/2018/6345412

**Published:** 2018-04-23

**Authors:** Stef De Lombaerde, Ken Kersemans, Sara Neyt, Jeroen Verhoeven, Christian Vanhove, Filip De Vos

**Affiliations:** ^1^Laboratory of Radiopharmacy, Ghent University, Ottergemsesteenweg 460, Ghent, Belgium; ^2^Ghent University Hospital, Department of Nuclear Medicine, De Pintelaan 185, Ghent, Belgium; ^3^IBiTech-MEDISIP-INFINITY, Ghent University, Ghent, Belgium

## Abstract

**Introduction:**

An* in vivo *determination of bile acid hepatobiliary transport efficiency can be of use in liver disease and preclinical drug development. Given the increased interest in bile acid Positron Emission Tomography- (PET-) imaging, a further understanding of the impact of 18-fluorine substitution on bile acid handling* in vitro *and* in vivo* can be of significance.

**Methods:**

A number of bile acid analogues were conceived for nucleophilic substitution with [^18^F]fluoride: cholic acid analogues of which the 3-, 7-, or 12-OH function is substituted with a fluorine atom (3*α*-[^18^F]FCA; 7*β*-[^18^F]FCA; 12*β*-[^18^F]FCA); a glycocholic and chenodeoxycholic acid analogue, substituted on the 3-position (3*β*-[^18^F]FGCA and 3*β*-[^18^F]FCDCA, resp.). Uptake by the bile acid transporters NTCP and OATP1B1 was evaluated with competition assays in transfected CHO and HEK cell lines and efflux by BSEP in membrane vesicles. PET-scans with the tracers were performed in wild-type mice (*n* = 3 per group): hepatobiliary transport was monitored and compared to a reference tracer, namely, 3*β*-[^18^F]FCA.

**Results:**

Compounds 3*α*-[^18^F]FCA, 3*β*-[^18^F]FGCA, and 3*β*-[^18^F]FCDCA were synthesized in moderate radiochemical yields (4–10% n.d.c.) and high radiochemical purity (>99%); 7*β*-[^18^F]FCA and 12*β*-[^18^F]FCA could not be synthesized and included further in this study.* In vitro *evaluation showed that 3*α*-FCA, 3*β*-FGCA, and 3*β*-FCDCA all had a low micromolar* Ki*-value for NTCP, OATP1B1, and BSEP.* In vivo, *3*α*-[^18^F]FCA, 3*β*-[^18^F]FGCA, and 3*β*-[^18^F]FCDCA displayed hepatobiliary transport with varying efficiency. A slight yet significant difference in uptake and efflux rate was noticed between the 3*α*-[^18^F]FCA and 3*β*-[^18^F]FCA epimers. Conjugation of 3*β*-[^18^F]FCA with glycine had no significant effect* in vivo*. Compound 3*β*-[^18^F]FCDCA showed a significantly slower hepatic uptake and efflux towards gallbladder and intestines.

**Conclusion:**

A set of ^18^F labeled bile acids was synthesized that are substrates of the bile acid transporters* in vitro *and* in vivo* and can serve as PET-biomarkers for hepatobiliary transport of bile acids.

## 1. Introduction

Bile acids are steroid derivatives that are produced by the hepatocytes of the liver and excreted in bile. These molecules play an important role in micelle formation for lipid digestion and uptake of fat-soluble vitamins in the intestines [1]. The majority of bile acids (95%) is then reabsorbed and transported back to the liver by the portal circulation. Bile acid homeostasis in the liver is maintained by specific bile acid transporters on the hepatocytes. Uptake at the basolateral side relies predominantly on the Na^+^-dependent Taurocholate Cotransporting Polypeptide (NTCP) and also the Organic Anion Transporting Polypeptides (OATPs). Once in the hepatocyte, excretion in the bile canaliculi is mainly mediated by the Bile Salt Export Pump (BSEP) [2].

However, this highly efficient hepatobiliary transport of bile acids can be disturbed by xenobiotics that inhibit the aforementioned bile acid transporters or in certain liver diseases such as primary biliary cirrhosis or progressive familial intrahepatic cholestasis (PFIC) [3–5]. A toxic build-up of bile acids in the hepatocytes, termed cholestasis, can then present itself. Clinical features may consist of nausea, abdominal pain, jaundice and pruritus [6]. An accurate* in vivo *determination of bile acid transport efficiency can therefore be valuable for detection or evaluation of liver disease in both drug development and clinic.

To visualize physiological processes on a molecular level* in vivo*, Positron Emission Tomography (PET) is the imaging modality of choice. The possibility for close resemblance of the PET-radiotracer and the endogenous substrate under investigation gives this imaging technique an important asset [7]. Recently, a number of studies with ^11^C or ^18^F labeled bile acid PET-tracers have been published. Frisch et al. evaluated ^11^C labeled bile acids, conjugated with sarcosine or N-methyl taurine in pigs [8, 9]. These tracers were able to visualize hepatobiliary transport and can provide valuable information in hepatobiliary diseases, although the short half-life of the ^11^C isotope limits their use. Several ^18^F labeled bile acids were developed to overcome this issue. Jia et al. developed a ^18^F labeled bile acid for studying Farnesoid X Receptor- (FXR-) related diseases, using a click reaction of 1,3-dipolar cycloaddition of terminal alkynes and organic azides [10]. However, this modification removes the terminal carboxylic acid moiety that is critical for recognition by the bile acid transporters [11]. Testa et al. improved this click chemistry approach by retaining the carboxylic acid functional group [12]. Nevertheless, for both ^18^F labeled compounds the introduction of a long 1,2,3-triazole linked fluoroalkyl sidechain on the carboxylic acid terminus encompasses a substantial addition to the steroid structure. This might result in altered hepatobiliary transport, compared to endogenous bile acids.

Because of the need for a ^18^F labeled bile acid with only a minimal modification in molecular structure compared to an endogenous bile acid, 3*β*-[^18^F]fluorocholic acid (3*β*-[^18^F]FCA) was developed by our research group [13]. The bile acid structure for 3*β*-[^18^F]FCA is cholic acid, of which the 3 alpha OH group was substituted for a 3-beta fluorine. This tracer showed transport by NTCP, OATP, and BSEP* in vitro *and could visualize hepatobiliary transport* in vivo* and drug-induced alterations thereof.

Given the increased interest in bile acid PET-imaging, a further understanding of the impact of fluorine-OH substitution on bile acid handling* in vitro *and* in vivo* can be beneficial. Therefore, in this study a number of fluorinated analogues of cholic, chenodeoxycholic, and glycocholic acid were synthesized, evaluated, and compared* in vitro *and* in vivo *in mice.

## 2. Materials and Methods

### 2.1. Radiosynthesis of the ^18^F-Labeled Bile Acids

**Figure 1:**
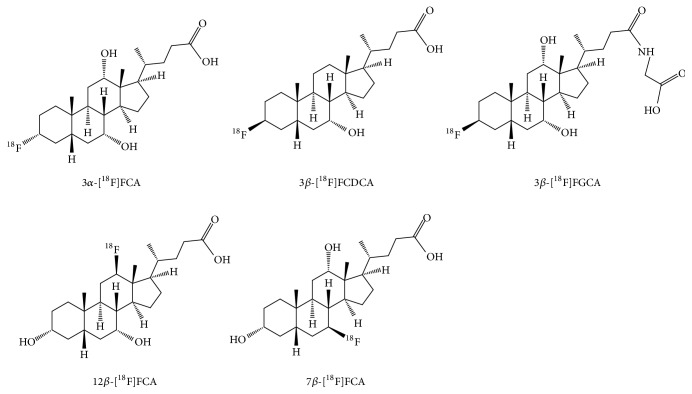
^18^F labeled bile acid analogues under investigation in the present study. Compounds 3*α*-[^18^F]fluorocholic acid (3*α*-[^18^F]FCA), 3*β*-[^18^F]fluorochenodeoxycholic acid (3*β*-[^18^F]FCDCA), and 3*β*-[^18^F]fluoroglycocholic acid (3*β*-[^18^F]FGCA) on the upper row differ in bile acid structure (cholic acid, chenodeoxycholic acid, and glycocholic acid) or 3 *α*/*β* position of the radiolabel. The structures 12*β*-[^18^F]fluorocholic acid (12*β*-[^18^F]FCA) and 7*β*-[^18^F]fluorocholic acid (7*β*-[^18^F]FCA) have the same cholic acid structure, but different position of the radiolabel (7 or 12).

The ^18^F-isotope was introduced on the bile acid skeleton by a nucleophilic substitution on a suitable mesylate, protected precursor molecule of which the synthesis can be found in Supplementary Data (available ). The conceived ^18^F labeled bile acids are shown in Figure 1. The radiosynthesis was performed as described earlier [13]. In short, [^18^F]fluoride (1.3 GBq) was trapped on a Sep-Pak QMA-column (Waters, Zellik, Belgium) that was preconditioned with 5 mL 0.01 M K_2_CO_3_ and 5 mL ultrapure water. The activity was eluted in a radiosynthesis vial with 1 mL 9 : 1 AcN : H_2_O Cryptand-2.2.2 (Acros Organics, Geel, Belgium) and K_2_CO_3_ (20 mg and 2 mg, resp.) solution. The solvents were removed by evaporation under a gentle nitrogen flow at 100°C. The residue was dried further by adding and evaporating 2 × 500 *μ*L AcN.

The precursor for radiosynthesis was dissolved in 200 *μ*L anhydrous DMSO and added to the radiosynthesis vial. A fixed amount (6.84 *μ*mol; 3.4–4.4 mg) of precursor for 3*α*-[^18^F]fluorocholic acid (3*α*-[^18^F]FCA), 3*β*-[^18^F]fluorocholic acid (3*β*-[^18^F]FCA), 7*β*-[^18^F]fluorocholic acid (7*β*-[^18^F]FCA), 12*β*-[^18^F]fluorocholic acid (12*β*-[^18^F]FCA), 3*β*-[^18^F]fluoroglycocholic acid (3*β*-[^18^F]FGCA), and 3*β*-[^18^F]fluorochenodeoxycholic acid (3*β*-[^18^F]FCDCA) was used. The vial was sealed, shaken, and heated for 20 minutes at 120°C. Afterwards, the vial was cooled and 100 *μ*L 5 M NaOH was added. The mixture was shaken and heated again at 120°C for 10 minutes. After cooling and neutralization of the basic reaction mixture, purification was performed by a semipreparative HPLC-system (Grace Econosphere C18 10.0 × 250 mm, 10 *μ*m; 6 mL/min 10% AcN in H_2_O -> 100% AcN in 20 minutes; radiodetection (Ludlum Measurements Inc.)). The desired HPLC-fraction was collected, diluted with ultrapure water to 50 mL, and loaded on a Sep-Pak C18 Plus short cartridge (preconditioned with 10 mL EtOH and 10 mL ultrapure water). After washing with 10 mL ultrapure water, the ^18^F-labeled bile acid was eluted off the column with 1 mL EtOH in a separate vial. This fraction was evaporated under a gentle N_2_-flow and heating. Finally, 500 *μ*L phosphate buffered saline was added to reformulate the tracer for* in vivo *use.

The log*D*-value, stability (in formulation for use and mouse serum), and identity of the tracer were assessed as described earlier [13]. Radiochemical purity and percentage ^18^F-labeling of precursor were determined by radio-TLC (silica gel TLC; with 10% MeOH in CH_2_Cl_2_ and 4 : 1 AcN : H_2_O, resp.).

### 2.2. In Vitro Uptake Assays

Transport of the fluorinated bile acids by NTCP and OATP1B1 was evaluated by their inhibition of [^3^H]taurocholate ([^3^H]TC; Perkin Elmer) and [^3^H]estradiol-17*β*-glucuronide ([^3^H]EbG; Perkin Elmer) uptake, respectively. Cholic acid (CA) was added to the test compound panel as a reference. Chinese Hamster Ovary (CHO) and Human Embryonic Kidney (HEK) cells expressing NTCP and OATP1B1, respectively (Solvo Biotechnologies), were used. Maintenance of these cell cultures was described earlier [13].* Ki*-values of the different bile acids were calculated using the experimentally determined IC50-and* Km*-parameters in the Cheng-Prusoff equation (GraphPad Prism v3.00 Software) (see (1))

(1) $$\begin{array}{c} Ki=\frac{IC50}{1+\left[S\right]/Km}, \end{array}$$

where *Ki* refers to inhibition constant; IC50 refers to concentration of fluorinated bile acid that causes a 50% decrease in ^3^H labeled substrate uptake; [*S*] refers to concentration of ^3^H labeled substrate;* Km* refers to Michaelis-Menten constant of ^3^H labeled substrate

Cells were seeded in 24-well plates at 400,000 cells/well. The culture medium was aspirated after 24 hours and the cells were washed twice with 1 mL 10 mM HEPES-Hank's Balanced Salt Solution (HBSS; with Ca and Mg; 37°C, pH 7). For* Km*-determination, cells were incubated with 250 *μ*L of 0–150 *μ*M [^3^H]TC (NTCP) or [^3^H]EbG (OATP1B1) for 10 minutes at 37°C. IC50-values were acquired by incubating the cells with 250 *μ*L 0–1000 *μ*M fluorinated bile acid and 1 *μ*M [^3^H]TC (NTCP) or [^3^H]EbG (OATP1B1) for 15 minutes at 37°C. All dosing solutions were formulated in the washing buffer and dosing was executed in triplicate. The incubation was stopped by placing the plates on ice and adding 1 mL ice-cold 1% BSA HBSS-solution. The supernatant was aspirated and the cells were washed twice with 2 mL ice-cold HBSS-solution. NaOH (250 *μ*l, 0.1 M) was added to lyse the cells and the plates were shaken (15 minutes, 37°C). Aliquots of this lysate were subjected to liquid scintillation counting (TriCarb 2900 TR; Perkin Elmer) and protein determination with a BCA-assay (ThermoFisher Scientific).

### 2.3. In Vitro Efflux Assays

Transport of the bile acids by BSEP was evaluated by their inhibition of [^3^H]TC uptake in BSEP membrane vesicles (Pharmtox). Cholic acid (CA) was added to the test compound panel as a reference.* Ki*-values of the different bile acids were calculated using the experimentally determined IC50-and* Km*-parameters in the Cheng-Prusoff equation (GraphPad Prism v3.00 Software) (see (1)). The assay was performed in V-tip 96-well plates; each well contained 3.75 *μ*g membrane vesicle, 10 mM MgCl_2_, 10 mM Tris, 4 mM adenosine triphosphate (ATP), and 250 mM sucrose. A concentration range of 0–50 *μ*M [^3^H]TC was included for the* Km*-determination. To determine the IC50s, 0–2500 *μ*M of bile acid was used with 0.65 *μ*M [^3^H]TC. All dosing was performed in triplicate. The plates were suspended in a 37°C water bath 1 minute before the incubation. Incubation was started by adding 10 *μ*L of the ATP solution or buffer solution (negative control). Each well contained a final volume of 30 *μ*L and the plates were incubated for 10 minutes at 37°C.

Transport was stopped by placing the plates on ice and adding 150 *μ*L ice-cold buffer solution. The contents of each well were pipetted in a glass fiber filter plate (Multiscreen HTS plates; Merck Millipore) and washed 3 times with 200 *μ*L ice-cold buffer solution. Finally, 100 *μ*L 0.1 M NaOH was added: lysis of the vesicles took place for 10 minutes at room temperature. An aliquot was used for liquid scintillation counting.

### 2.4. PET-Imaging Protocol

The* in vivo* transport characteristics of the ^18^F labeled bile acids were evaluated in wild-type FVB-mice (female, 5 w). The tracer described earlier [13], 3*β*-[^18^F]FCA, was also included as a reference standard. Imaging was performed with a PET/CT (FLEX Triumph II small animal PET/CT-scanner; axial field of view: 7.5 cm; 1.3 mm spatial resolution; TriFoil Imaging). For each tracer, three animals were used. The animals were housed and handled in accordance with the European Ethics Committee guidelines and the experiments were approved by the Animal Experimental Ethical Committee of Ghent University (ECD 15/69).

Food and water were provided ad libitum, but the animals were fasted overnight before a PET/CT-scan. They were anesthetized with 1.5 v : v% isoflurane in O_2_ and placed on a heated bed. To allow injection of the tracer, a polyethylene intravenous line was inserted in the lateral tail vein and fixed. After the animals were transferred to the scanner animal bed, a 1 hour PET-scan was started and 9 MBq tracer was injected directly after starting the scan. Following this PET-scan, 9 MBq [^18^F]FDG was injected and twenty minutes later, a second PET-scan with a 20-minute acquisition time was started.

All PET-scans were obtained in list-mode and were iteratively reconstructed (50 iterations). The 1-hour scan was reconstructed in the following frames: 8 × 15 s; 16 × 30 s; 10 × 60 s; 20 × 120 s. For presentation purposes, Maximum Intensity Projection PET/CT images were generated in Amide software. The data were analyzed using Pmod software v3.405 (PMOD Technologies): Regions Of Interest (ROIs) were drawn manually over the liver, gallbladder, and intestines. On the static [^18^F]FDG scan, the left ventricle was delineated and this ROI was pasted on the dynamic scan to obtain an image-derived arterial blood concentration. The uptake of radioactivity in liver, gallbladder, and intestines was expressed as a percentage of the injected dose (% ID) and normalized for the weight of a 20 g mouse. The % ID in these organs was monitored in function of time to obtain time-activity curves (TACs). Biliary clearance of the tracers was determined with equation 2 from Ghibellini et al. [14]

(2) Biliary  clearance=cumulative  amount  of  tracer  in  gallbladder&intestinesAUC  blood  concentration0→60 min.

The Area Under the Curve (AUC), % ID, and time-to-peak values of the TACs were determined in GraphPad Prism v3.00 Software. The obtained parameters of the different ^18^F labeled bile acids were compared to 3*β*-[^18^F]FCA-values in SPSS Statistics 23 Software. Differences between two groups were analyzed with the nonparametric Mann–Whitney* U* test. A *p* value ≤ 0.05 was considered significant.

## 3. Results

### 3.1. Radiosynthesis of the ^18^F-Labeled Bile Acids

Differences in radiolabeling yield of the precursors for radiosynthesis with [^18^F]fluoride were observed (Table 1). The precursors for 3*α*-[^18^F]FCA and 3*β*-[^18^F]FCDCA had approximately the same labeling yield (30%) and nondecay corrected yield (10%). The precursor for 3*β*-[^18^F]FGCA was only labeled for 11.51 ± 2.55%, which also resulted in a lower total yield of 4.28 ± 0.39%. The tracers 3*α*-[^18^F]FCA, 3*β*-[^18^F]FCDCA, and 3*β*-[^18^F]FGCA were found to be stable in its formulation and in mouse serum. Both the precursors for 12*β*-[^18^F]FCA and 7*β*-[^18^F]FCA provided minimal radiolabeling: generating a significant amount of completed product proved troublesome for these two products. Furthermore, the 7*β*FCA reference compound could not be synthesized. The Log*D* values of the ^18^F labeled bile acids are displayed in Table 1.

### 3.2. In Vitro Uptake Assays

**Figure 2:**
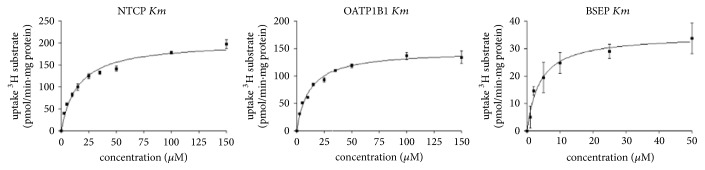
Graphs of the concentration dependent uptake of [^3^H]TC in CHO-NTCP cells and BSEP membrane vesicles. [^3^H]EbG was used in HEK-OATP1B1 cells. All data are mean ± SD (*n* = 3).

A Michaelis-Menten curve was made for [^3^H]TC and [^3^H]EbG for the CHO-NTCP and HEK-OATP1B1 cells, respectively (Figure 2). For the CHO-NTCP cells, the* Km* of [^3^H]TC was 15.27 ± 2.46 *μ*M and the* V*max 200.4 ± 9.77 pmol/min·mg protein. For the HEK-OATP1B1 cells, the* Km* of [^3^H]EbG was 11.72 ± 1.59 *μ*M and the* V*max 146.4 ± 5.52 pmol/min·mg protein.

**Figure 3:**
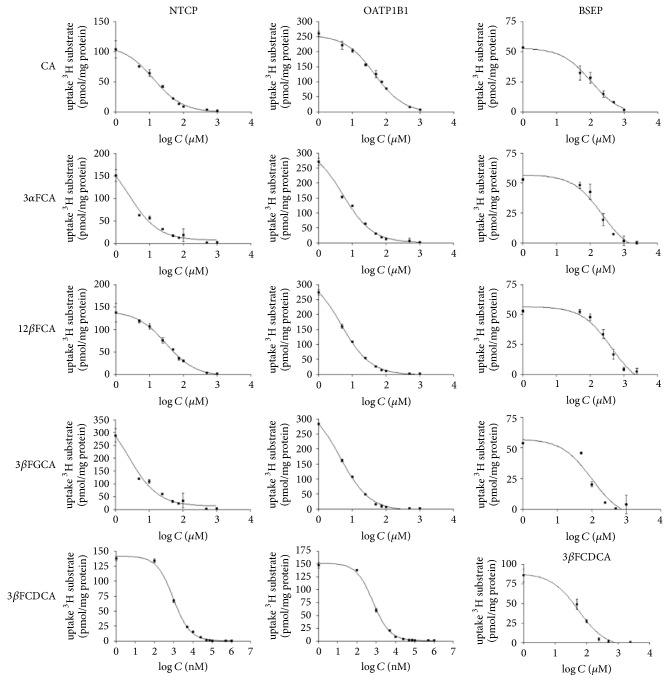
Graphs of the concentration dependent decrease in uptake of tritium labeled substrate ([^3^H]TC for NTCP and BSEP; [^3^H]EbG for OATP1B1). IC50s of the compounds were determined by GraphPad, and their respective* Ki*-values can be found in Table 2. All data are mean ± SD (*n* = 3).

Cholic acid and the fluorinated bile acid analogues caused a dose-dependent decrease in the uptake of [^3^H]TC and [^3^H]EbG for NTCP and OATP1B1. A sigmoidal dose-response curve was fitted on the acquired data points to determine the IC50 and calculate the* Ki*-value (Figure 3 and Table 2).

### 3.3. In Vitro Efflux Assays

A Michaelis-Menten curve of [^3^H]TC was made for BSEP (Figure 2). The* Km* of [^3^H]TC was 3.89 *μ*M ± 1.44 *μ*M and the* V*max 34.97 ± 3.57 pmol/min·mg protein. Cholic acid and the fluorinated bile acid analogues caused a dose-dependent decrease in the uptake of [^3^H]TC in the BSEP membrane vesicles. A sigmoidal dose-response curve was fitted on the acquired data points to determine the IC50 and calculate the* Ki*-value (Figure 3 and Table 2).

### 3.4. In Vivo Evaluation of the ^18^F-Labeled Bile Acids

**Figure 4:**
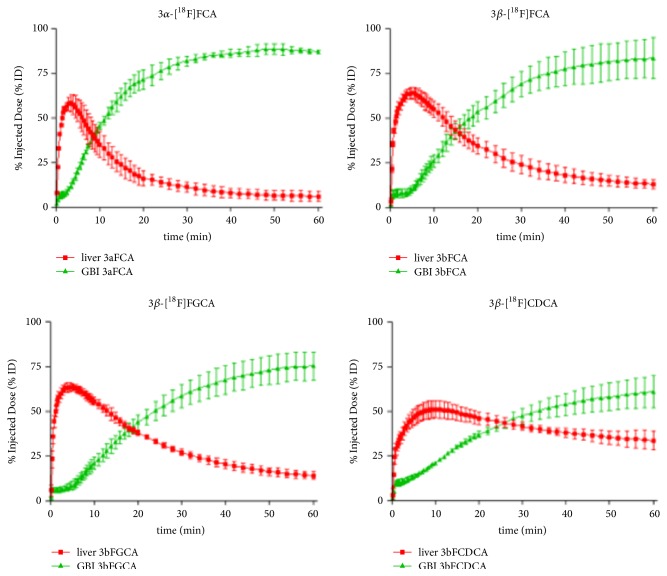
Time-activity curves (TACs) of 3*α*-[^18^F]FCA, 3*β*-[^18^F]FCA, 3*β*-[^18^F]FGCA, and 3*β*-[^18^F]FCDCA in liver (red curve) and gallbladder and intestines (GBI; green curve) of wild-type mice. Uptake of the tracers was expressed as % injected dose (% ID) and normalized for a 20 g mouse. Data are mean ± SD (*n* = 3 per group).

**Figure 5:**
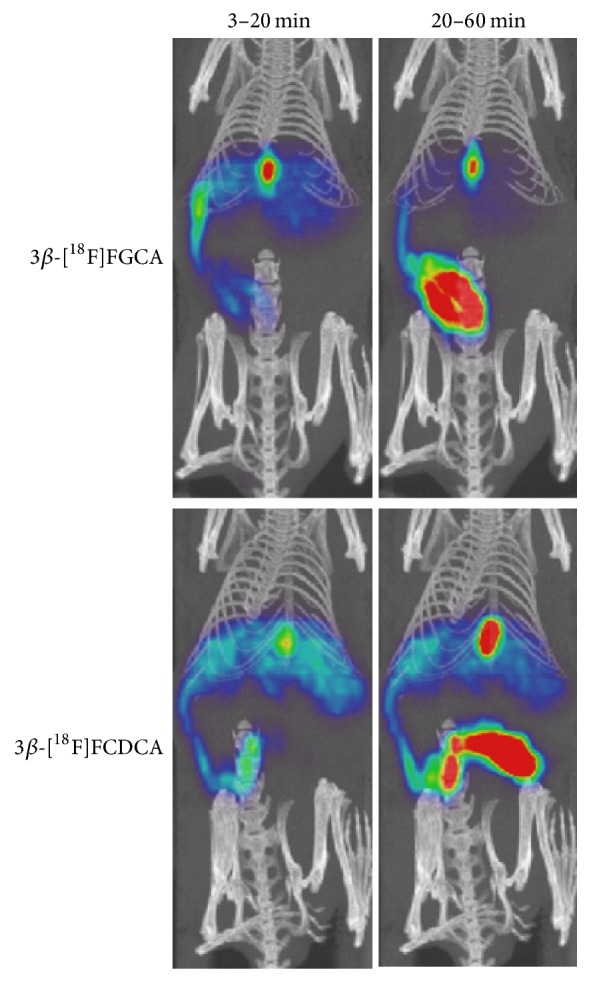
Representative Maximum Intensity Projection PET/CT of 9 MBq 3*β*-[^18^F]FGCA and 3*β*-[^18^F]FCDCA in a wild-type mouse (slice thickness: 12 mm). After intravenous injection, both tracers showed exclusive uptake in the liver (3–20 minutes). The ^18^F labeled bile acids were then excreted in gallbladder and intestines. Biliary excretion of 3*β*-[^18^F]FCDCA is visually slower than 3*β*-[^18^F]FGCA (20–60 minutes after injection).

The ^18^F labeled bile acids 3*α*-[^18^F]FCA, 3*β*-[^18^F]FCA, 3*β*-[^18^F]FGCA, and 3*β*-[^18^F]FCDCA were evaluated in wild-type mice. Because of their negligible radiochemical yield, 7*β*-[^18^F]FCA and 12*β*-[^18^F]FCA could not be included. Compounds 3*β*-[^18^F]FCA, 3*α*-[^18^F]FCA, 3*β*-[^18^F]FGCA, and 3*β*-[^18^F]FCDCA showed exclusive hepatic uptake after intravenous injection. The hepatic uptake of reference tracer 3*β*-[^18^F]FCA was significantly slower than 3*α*-[^18^F]FCA (5.33 ± 0.24 versus 3.33 ± 0.62 minutes), but significantly faster than 3*β*-[^18^F]FCDCA (5.33 ± 0.24 versus 10.17 ± 0.62 minutes). There was no difference in the time-to-peak of the liver TAC of 3*β*-[^18^F]FCA and 3*β*-[^18^F]FGCA. Once in the liver, the tracers were excreted in gallbladder and intestines. Almost all of the injected activity (approx. 80%) of 3*α*-[^18^F]FCA, 3*β*-[^18^F]FCA, and 3*β*-[^18^F]FGCA was found in gallbladder and intestines after 1 hour. Due to slower excretion of 3*β*-[^18^F]FCDCA from the liver, only 60% of the injected dose was found in gallbladder and intestines after 1 hour. Of the four tracers, only the biliary clearance of 3*β*-[^18^F]FCDCA (0.18 ± 0.04 mL/min) was significantly different compared to 3*β*-[^18^F]FCA (0.46 ± 0.08 mL/min). No radioactivity was observed in other organs for all tracers under investigation. Time-activity curves of the ^18^F labeled bile acids were generated and the relevant parameters were extracted from these graphs (Figure 4 and Table 3). Representative PET/CT images of 3*β*-[^18^F]FGCA and 3*β*-[^18^F]FCDCA are displayed in Figure 5.

## 4. Discussion

Cholestasis, a toxic accumulation of bile acids in the liver or blood, may occur in certain liver diseases or can be triggered as a result of a xenobiotic interfering with the hepatobiliary transport of bile acids [15]. It is therefore important to have an adequate tool to evaluate the efficiency of bile acid transport* in vivo* for clinical use or in drug development.

To that end, PET-imaging of hepatobiliary transport with radiolabeled bile acids is gaining importance. A number of studies have been published in which bile acids were labeled with either ^11^C or ^18^F on different bile acid structures [8–10, 12]. Our research group developed 3*β*-[^18^F]fluorocholic acid (3*β*-[^18^F]FCA), a cholic acid derivative of which the 3*α*-OH function is substituted for a 3*β*-fluorine atom. This minimal modification of the endogenous molecule allowed visualization and quantification of bile acid hepatobiliary transport and drug-induced alterations thereof. Seeing the promising results with 3*β*-[^18^F]FCA and the increased interest in hepatobiliary imaging with PET, a further exploration of fluorinated bile acid transport characteristics was performed in this study. A number of fluorinated analogues were conceived: cholic acid derivatives of which the 3, 7, and 12 OH functions were replaced with a fluorine atom, a 3*β*-fluorine labeled chenodeoxycholic acid, and 3*β*-fluorine labeled glycocholic acid. These compounds were evaluated on their radiosynthesis characteristics,* in vitro* and* in vivo *hepatobiliary transport in wild-type mice.

Of the conceived ^18^F labeled bile acids, only 3*α*-[^18^F]FCA, 3*β*-[^18^F]FGCA, and 3*β*-[^18^F]FCDCA could be obtained in a suitable radiochemical yield. The ^18^F labeling yield of the precursor molecules for 3*α*-[^18^F]FCA and 3*β*-[^18^F]FCDCA was comparable to that of 3*β*-[^18^F]FCA described earlier (approx. 30%) [13]. Labeling of the 3*β*-[^18^F]FGCA precursor was only 11.51 ± 2.55%. This reduced yield could be attributed to the presence of a more polar amide group on the precursor, which can reduce the [^18^F]fluoride nucleophilic substitution efficiency. Log*D* values of the three tracers mentioned above were determined and were in line with literature Log*D* values of cholic acid, chenodeoxycholic acid, and glycocholic acid [16].

The mesylate precursors for 12*β*-[^18^F]FCA and 7*β*-[^18^F]FCA could not be substituted efficiently with [^18^F]fluoride and could therefore not be included in the* in vivo *evaluation. The 7*α*- and 12*α*- mesyl groups on cholic acid are positioned axially on steroid rings B and C, respectively. Coupled to the presence of neighboring hydrogen atoms that can be removed by a base, these positions are more prone to elimination than substitution with [^18^F]fluoride. This observation was also made in the attempted synthesis of the 12*β*-[^18^F]FCA and 7*β*-[^18^F]FCA reference compounds. The 7*β*FCA synthesis with DAST resulted exclusively in the formation of elimination product. However, it was possible to synthetize the 12*β*-FCA reference compound in low yield, as there are less protons available for elimination by a base due to the 19-methyl group present near the 12*α*-mesyl-group.* In vitro *data for 12*β*-FCA could be acquired, but no* in vivo* PET-imaging was possible.

*Ki*-values of CA, 3*α*FCA, 3*β*FGCA, 3*β*FCDCA, and 12*β*FCA for the bile acid transporters NTCP, OATP1B1, and BSEP were determined as an affinity measure. It was demonstrated that these compounds caused a concentration dependent decrease in uptake of the tritium labeled model substrates of NTCP, OATP1B1, and BSEP. The substitution of the 3-OH function by a more lipophilic fluorine atom on the 3*α*- and 3*β*-position of CA causes an increase in affinity for NTCP and OATP1B1, yet gives rise to a decrease in affinity for the BSEP-transporter compared to CA. Although both 3-FCA epimers are a substrate for the bile acid transporters, 3*α*-FCA displays a slightly higher affinity than 3*β*-FCA for NTCP and OATP1B1 (2.53 ± 0.76 *μ*M and 5.22 ± 0.56 *μ*M, resp., versus 6.18 ± 0.59 and 9.67 ± 0.83 *μ*M, resp.). The affinity for BSEP does not change. This difference in affinity for 3 *α*/*β* cholic acid epimers of bile acid transporters was already uncovered in cell lines that express the Apical Sodium-dependent Bile acid Transporter (ASBT; responsible for basolateral uptake of bile acids in the enterocytes). It was found that 3*β*-OH-bile acids have a lower affinity than 3*α*OH-bile acids for ASBT [17]. In the present study, the hepatic basolateral uptake transporters NTCP and OATP1B1 also reveal a slight preference in affinity for the 3*α*-fluorocholic acid epimer. Compound 3*β*-FGCA, 3*β*-cholic acid conjugated with the amino acid glycine, showed an increase in affinity for all bile acid transporters under investigation. This is in line with literature data: conjugated bile acids have a higher affinity for NTCP, OATP, and BSEP than nonconjugated bile acids [2, 18].

The PET-scans with 3*β*-[^18^F]FCA, 3*α*-[^18^F]FCA, 3*β*-[^18^F]FGCA, and 3*β*-[^18^F]FCDCA showed hepatobiliary transport after intravenous injection in healthy wild-type FVB-mice. The former three tracers displayed fairly similar TAC-curves, whereas 3*β*-[^18^F]FCDCA had the most aberrant curves. Compared to 3*β*-[^18^F]FCA, there is a significantly slower liver uptake from the blood compartment: both the time-to-peak of the liver TAC and the AUC of tracer in arterial blood increased approximately twofold. Furthermore, a significant drop in excretion towards gallbladder and intestines was observed. This slower hepatobiliary clearance of 3*β*-[^18^F]FCDCA* in vivo, *although coupled with the* in vitro *observation that this fluorinated bile acid has a very high affinity for the bile acid transporters, implies that 3*β*FCDCA acts as slow substrate for the bile acid transporters* in vivo*. Because 3*β*-[^18^F]FCDCA already shows slower hepatobiliary transport in healthy wild-type mice, this tracer is less suited to detect possible alterations of bile acids transport by pharmacological interference or in liver disease.

Considering the 3*α*/*β*-[^18^F]FCA epimers, it was found that 3*α*-[^18^F]FCA shows a slight, yet significant decrease in time-to-peak of the liver TAC and excretion to gallbladder and intestines proceeded faster. This is reflected in a decrease of the liver TAC AUC-value and modest increase in AUC of the gallbladder and intestines TAC compared to 3*β*-[^18^F]FCA. These results illustrate that there is not only an observable difference in affinity of the 3*α*/*β* FCA epimers for bile acid transporters* in vitro*, but also in hepatobiliary transport* in vivo*. There is a slight preference for 3*α*-[^18^F]FCA compared to 3*β*-[^18^F]FCA, probably because the fluorine atom is in the same 3*α*-configuration as the 3*α*-OH on the endogenous cholic acid molecule.

Conjugation of 3*β*-[^18^F]FCA with glycine did not have a significant impact on its* in vivo *hepatobiliary transport, although the affinity of 3*β*FGCA for NTCP, OATP1B1, and BSEP rises compared to 3*β*FCA* in vitro*. This means that glycine conjugation of a bile acid is not a prerequisite for faster or more efficient hepatobiliary transport in mice. The majority of the murine bile acid spectrum is however composed of taurine conjugated bile acids [19]. The observed identical transport efficiency of 3*β*-[^18^F]FCA and its glycine conjugate 3*β*-[^18^F]FGCA could be different if taurine conjugation were explored.

Two out of three newly developed tracers (3*α*-[^18^F]FCA and 3*β*-[^18^F]FGCA) display fast and efficient hepatobiliary transport and can therefore be employed as biomarker for bile acid transport* in vivo*. Given the observed discrepancy between* in vitro *affinity and the* in vivo *hepatobiliary transport of some bile acid transporter substrates (such as 3*β*FGCA and 3*β*FCDCA), it is still important to determine the* in vivo* kinetics of a bile acid transporter substrate and not to rely on* in vitro *results alone to assess potentially disturbed hepatobiliary transport of bile acids.

## 5. Conclusion

A set of ^18^F labeled bile acids were synthesized in a moderate radiochemical yield: 3*α*-[^18^F]FCA, 3*β*-[^18^F]FGCA, and 3*β*-[^18^F]FCDCA. All tracers were* in vitro *substrates of the bile acid transporters NTCP, OATP1B1, and BSEP and showed hepatobiliary transport* in vivo*. It was found that 3*α*-[^18^F]FCA shows slightly faster hepatobiliary transport than its epimer, 3*β*-[^18^F]FCA. Conjugation of 3*β*-[^18^F]FCA with glycine is not a prerequisite for faster transport. The ^18^F labeled bile acids 3*α*-[^18^F]FCA and 3*β*-[^18^F]FGCA can be applied as biomarker for* in vivo *PET monitoring of hepatobiliary transport of bile acids. The ^18^F-chenodeoxycholic acid derivative, however, 3*β*-[^18^F]FCDCA, suffers from slower hepatobiliary transport than its cholic acid counterpart and is hence less suited for monitoring possible disturbances of bile acid transport* in vivo*.

## Figures and Tables

**Table 1:** Radiosynthesis characteristics of the different ^18^F labeled bile acids. The overall synthesis time was 100 minutes. n.d.c. RY: nondecay corrected radiochemical yield. RP: radiochemical purity. Data are mean ± SD.

	n.d.c. RY (%) (*n* = 3)	^18^F labeling of precursor (%) (*n* = 3)	RP (%)	Log*D* (*n* = 3)
3*α*-[^18^F]FCA	10.56 ± 2.02	30.31 ± 1.28	>99	0.92 ± 0.17
3*β*-[^18^F]FGCA	4.28 ± 0.39	11.51 ± 2.55	>99	0.011 ± 0.037
3*β*-[^18^F]FCDCA	9.57 ± 1.51	28.27 ± 5.02	>99	1.42 ± 0.16
12*β*-[^18^F]FCA	<0.5	2.82 ± 1.14	NA	NA
7*β*-[^18^F]FCA	<0.5	1.23 ± 0.26	NA	NA

**Table 2:** Calculated *Ki*-values of the bile acid analogues for NTCP, OATP1B1, and BSEP. Data are mean ± SD (*n* = 3). ^*∗*^3*β*FCA IC50-values were extracted from literature [13] and used to calculate the *Ki*-values.

	*Ki* for NTCP (*µ*M)	*Ki* for OATP1B1 (*µ*M)	*Ki* for BSEP (*µ*M)
CA	12.96 ± 4.26	42.70 ± 0.16	95.46 ± 33.97
3*α*FCA	2.53 ± 0.76	5.22 ± 0.56	198.93 ± 39.47
3*β*FCA^*∗*^	6.18 ± 0.59	9.67 ± 0.83	216.30 ± 52.99
3*β*FGCA	1.09 ± 0.34	4.24 ± 0.16	87.38 ± 14.61
3*β*FCDCA	0.91 ± 0.02	0.64 ± 0.07	48.24 ± 3.94
12*β*FCA	30.08 ± 9.40	4.56 ± 0.25	367.02 ± 38.11

**Table 3:** Metrics of the ^18^F labeled bile acids time-activity curves. The values are expressed as mean ± SD (*n* = 3). Significant differences compared to the 3*β*-[^18^F]FCA values are marked with *∗*. A *p* value ≤ 0.05 was considered significant. GBI = gallbladder and intestines; AB = arterial blood.

	3*β*-[^18^F]FCA	3*α*-[^18^F]FCA	3*β*-[^18^F]FGCA	3*β*-[^18^F]FCDCA
AUC liver (% ID·min)	1785 ± 194	1065 ± 171^*∗*^	1907 ± 58	2449 ± 111^*∗*^
Max% ID liver (%)	63.80 ± 2.61	58.58 ± 3.19	63.58 ± 1.84	51.01 ± 3.94^*∗*^
Time-to-peak liver (min)	5.33 ± 0.24	3.33 ± 0.62^*∗*^	5.17 ± 0.62	10.17 ± 0.62^*∗*^
AUC GBI (% ID·min)	3499 ± 340	4184 ± 109^*∗*^	3021 ± 276	2515 ± 189^*∗*^
Max% ID GBI (%)	83.44 ± 9.27	88.70 ± 2.38	75.32 ± 6.30	60.93 ± 7.29^*∗*^
AUC AB concentration (MBq·min/mL)	13003 ± 1032	14007 ± 2150	10192 ± 1404	28437 ± 4380^*∗*^
Biliary clearance (mL/min)	0.46 ± 0.08	0.49 ± 0.10	0.47 ± 0.07	0.18 ± 0.04^*∗*^

## Supplementary Materials

Supplementary Materials The file “Supplementary Data” contains the organic synthesis pathways to obtain the precursors for radiosynthesis of 3*α*-[^18^F]FCA, 7*β*-[^18^F]FCA; 12*β*-[^18^F]FCA, 3*β*-[^18^F]FGCA, and 3*β*-[^18^F]FCDCA.
