# Supplementary material for: Evaluating Hepatobiliary Transport with 18F-Labeled Bile Acids: The Effect of Radiolabel Position and Bile Acid Structure on Radiosynthesis and In Vitro and In Vivo Performance
Source: Contrast Media Mol Imaging. 2018 Apr 23;2018:6345412. doi: 10.1155/2018/6345412 (PMC5941726; doi:10.1155/2018/6345412)
Supplement: Supplementary Materials — The file “Supplementary Data” contains the organic synthesis pathways to obtain the precursors for radiosynthesis of 3α-[18F]FCA, 7β-[18F]FCA; 12β-[18F]FCA, 3β-[18F]FGCA, and 3β-[18F]FCDCA. [file 6345412.f1.docx]

# Supplementary data

## General

All used chemicals were bought from Sigma Aldrich (Bornem, Belgium) and were at least reagent grade. Cholic acid methyl ester was acquired from TCI Europe (Zwijndrecht, Belgium). All solvents were obtained from Acros (Geel, Belgium) and were at least of HPLC-grade. Reaction progress was monitored on silica gel TLC-sheets (silica gel 60; Sigma Aldrich, Bornem, Belgium). Bile acid analogues on the TLC-strip were visualized by staining with phosphoric acid.

^1^H NMR spectra were recorded with a Varian Mercury BB 300 MHz (Palo Alto, CA, USA). Mass spectra were obtained by ESI (+ or -) with a Waters LCT-premier XE, TOF high resolution mass spectrometer (Milford, MA, USA).

**Procedure A: introduction of acetyl protecting groups**

The acetylation of the free hydroxyl functions on a bile acid methyl ester was based on similar reactions described in literature [1,2]**.** In a roundbottom flask, 2 mmol of bile acid and 0.4 mmol 4-DMAP was dissolved in 10 mL of pyridine. Acetic anhydride (58 mmol; 5.5 mL) was added and the reaction mixture was stirred overnight under inert atmosphere at room temperature. The reaction mixture was then poured into 100 mL 6 M HCl. The aqueous phase was extracted three times with 100 mL EtOAc. The organic fractions were combined and washed with 300 mL 6 M HCl, brine and water, after which it was dried over Na_2_SO_4_, filtered and concentrated *in vacuo*. This crude reaction product was then subjected to silicagel column chromatography.

**Procedure B: partial deprotection of acetyl group**

The partial deprotection of the acetyl protecting group on the 3 position of the bile acid methyl ester was based on similar reactions described in literature [1,2]**.** The acetyl protected bile acid methyl ester (1 mmol) was dissolved in 7 mL MeOH and the solution was cooled to 0°C. A solution of AcCl (94 µL) in MeOH (3 mL) was slowly added. The mixture was stirred at room temperature until full consumption of the starting product (24-32 h). A saturated NaHCO_3_ solution (50 mL) was added to quench the reaction. The aqueous phase was extracted three times with 100 mL EtOAc. The organic phases were combined, dried over Na_2_SO_4_, concentrated *in vacuo*. The residue was further purified by silicagel column chromatography.

**Procedure C: mesylation**

The mesylation of the free OH-function of the bile acid analogue was based on similar reactions described in literature [2,3]. The partially deprotected bile acid methyl ester (0.3 mmol) was dissolved in anhydrous pyridine (5 mL) under inert atmosphere. The solution was cooled to 0°C and MsCl (70 µL; 0.9 mmol) was slowly added. The mixture was stirred at room temperature until the starting product was consumed. Then, the content of the flask was emptied in 50 mL 6 M HCl and extracted three times with 50 mL CH_2_Cl_2_. The organic phases were combined and washed with 150 mL 6 M HCl, brine and water. Following this washing step, the organic phase was dried over MgSO_4_ and concentrated *in vacuo*. The residue was further purified by silicagel column chromatography.

**Procedure D: fluorination with DAST**

The fluorination of a partially deprotected bile acid methyl ester was based on a similar reaction in literature [2]. The bile acid analogue (0.3 mmol) was dissolved in 10 mL anhydrous CH_2_Cl_2_ and DAST (79 µL; 0.6 mmol) was added slowly. The reaction mixture was allowed to stir for 15 minutes at room temperature, after which it was diluted with 40 mL CH_2_Cl_2_ and 50 mL saturated NaHCO_3_. The organic phase was washed with 50 mL brine and water, dried over MgSO_4_ and concentrated *in vacuo*. The residue was purified by silicagel column chromatography.

**Procedure E: alkaline deprotection**

The deprotection of the fluorinated bile acid analogue was carried out in alkaline environment. The bile acid (0.2 mmol) was dissolved in 10 mL 5 % KOH in MeOH solution and refluxed for 45 minutes. The solvent was evaporated and the residue was transferred to a separatory funnel with 50 mL 6 M HCl and 50 mL CH_2_Cl_2_. The aqueous phase was extracted twice more with 50 mL CH_2_Cl_2_. The combined organic phases were washed with 150 mL saturated NaHCO_3_, brine and water. Finally, the CH_2_Cl_2_ was dried over MgSO_4_ and concentrated *in vacuo*. No further purification was performed.

**Synthesis of DNBZ-CAME (2)**

The Mitsunobu esterification with 3, 5-dinitrobenzoic acid was based on a similar reaction described in literature [4]. Cholic acid methyl ester (5 g, 11.83 mmol) 3, 5-dinitrobenzoic acid (5 g, 23.57 mmol) and PPh_3_ (9 g, 34.31 mmol) were dissolved in anhydrous THF under inert atmosphere. The mixture was warmed to 50°C and DIAD (2.1 mL, 10.67 mmol) was slowly added. After 48 hours at 50°C, the solvent was removed by rotary evaporation. Methanol was added to the residue and the desired product was precipitated. The precipitate was washed with water and subjected to silica gel column chromatography to afford a slightly yellow solid (78.57 %). MS: 634.3326 [M+NH4]. ^1^H NMR (DMSO-d_6_; 300 MHz): 9.01 (1H, s); 8.87 (2H, s); 5.26 (1H, m); 4.20 (1H, m); 4,16 (1H, m); 3.78 (1H, s); 3.63 (1H, s); 3.56 (3H, s)

**Synthesis of 3βOH-CAME (3)**

This reaction was based on a similar reaction in the article of Gunasekara et al [5]. A solution was made of DNBZ-CAME (1 g, 1.622 mmol) in 10 mL THF. A 10 % NaOMe solution in methanol was added. The reaction mixture was stirred for 15 minutes at room temperature, after which the solvents were removed by rotary evaporation. The residue was redissolved in 100 mL EtOAc and was washed with 100 mL saturated NaHCO_3_-solution, brine and water. The organic layer was dried over Na_2_SO_4_ and finally evaporated to yield a white solid (100 %). MS: 440.3362 [M+NH4]. ^1^H NMR (DMSO-d_6_; 300 MHz): 4.84 (1H, m); 4.10 (1H, m); 4.02 (2H, m overlap); 3.76 (1H, s); 3.61 (1H, s); 3.55 (3H, s)

**Synthesis of 3β, 7α, 12α TriAc-CAME (4)**

Compound **4** was synthesized by subjecting **3** to procedure A, yielding a white solid (53.94 %). MS: 556.3700 [M+NH4]. ^1^H NMR (DMSO-d_6_; 300 MHz): 4.94 (1H, m); 4.88 (1H, m); 4.76 (1H, m); 3.55 (3H, s); 2.04 (3H, s); 1.99 (3H, s); 1.96 (3H, s)

**Synthesis of 3β OH, 7α, 12α DiAc-CAME (5)**

Compound **5** was synthesized by subjecting **4** to procedure B, yielding a white solid (96.84 %). MS: 551.3215 [M+FA-H]. ^1^H NMR (DMSO-d_6_; 300 MHz): 4.93 (1H, m); 4.75 (1H, m); 4.16 (1H, m); 3.81 (1H, m); 3.55 (3H, s); 2.03 (3H, s); 1.97 (3H, s)

**Synthesis of 3β Ms, 7α, 12α DiAc-CAME (6)**

Compound **6** was synthesized by subjecting **5** to procedure C, yielding a white solid (72.0 %). MS: 602.3379 [M+NH4]. ^1^H NMR (DMSO-d_6_; 300 MHz): 4.95 (1H, m); 4.89 (1H, m); 4.78 (1H, m); 3.55 (3H, s); 3.10 (3H, s); 2.04 (3H, s); 2.00 (3H, s)

**Synthesis of 3α F, 7α, 12α DiAc CAME (7)**

Compound 5 was subjected to procedure D, yielding a white solid (62.6 %). MS: 543.2856 [M+Cl]. ^1^H NMR (DMSO-d_6_; 300 MHz): 4.93 (1H, m); 4.80 (1H, m); 4.68 (1H, m); 3.54 (3H, s); 2.02 (3H, s); 1.92 (3H, s)

**Synthesis of 3α FCA (8)**

Compound 7 was subjected to procedure E, yielding a white solid (92.0 %). MS: 409.2757 [M-H]. ]. ^1^H NMR (DMSO-d_6_; 300 MHz): 11.93 (1H, s); 5.48 (2H, m overlap); 4.07 (1H, m); 3.77 (1H, s); 3.58 (1H, s);

**Synthesis of glycocholic acid methyl ester (10)**

Cholic acid (compound 9) was esterified with glycine methyl ester according to a literature procedure [6]. A white solid was obtained (100 %). MS: 480.3327 [M+H]. ^1^H NMR (DMSO-d_6_; 300 MHz): 8.21 (1H, s); 4.32 (1H, m); 4.09 (1H, m); 4.00 (1H, m); 3.77 (2H, m, overlap); 3.59 (3H, s)

**Synthesis of 3α, 7α, 12α** **TriAc glycocholic acid methyl ester (11)**

Compound 10 was subjected to procedure A, yielding a white solid (70.0 %). MS: 606.3627 [M+H]. ^1^H NMR (DMSO-d_6_; 300 MHz): 8.22 (1H, s); 4.96 (1H, m); 4.77 (1H, m); 4.44 (1H, m); 3.77 (2H, m); 3.59 (3H, s); 2.05 (3H, s); 1.98 (3H, s); 1.96 (3H, s)

**Synthesis of 3α OH 7α, 12α DiAc Glycocholic acid methyl ester (12)**

Compound 11 was subjected to procedure B, yielding a white solid (72.8 %). MS: 564.3547 [M+H]. ^1^H NMR (DMSO-d_6_; 300 MHz): 8.22 (1H, s); 4.94 (1H, m); 4.74 (1H, m); 4.47 (1H, m); 3.77 (2H, m); 3.59 (3H, s); 2.05 (3H, s); 1.99 (3H, s)

**Synthesis of 3α Ms 7α, 12α DiAc Glycocholic acid methyl ester (13)**

Compound 12 was subjected to procedure C, yielding a white solid (75.5 %). MS: 642.3317 [M+H]. ^1^H NMR (DMSO-d_6_; 300 MHz): 8.22 (1H, s); 4.96 (1H, m); 4.77 (1H, m); 4.41 (1H, m); 3.78 (2H, m); 3.59 (3H, s); 3.14 (3H, s); 2.05 (3H, s); 1.99 (3H, s)

**Synthesis of 3β F 7α, 12α DiAc Glycocholic acid methyl ester (14)**

Compound 12 was subjected to procedure D, yielding a white solid (63.0 %). MS: 566.3504 [M+H]. ^1^H NMR (DMSO-d_6_; 300 MHz): 8.19 (1H, s); 4.95 (1H, m); 4.75 (2H, m overlap); 3.77 (2H, d); 3.59 (3H, s); 2.03 (3H, s); 1.99 (3H, s)

**Synthesis of 3β F glycocholic acid (15)**

Compound 14 was subjected to procedure E, yielding a white solid (75.5%). MS: 468.3135 [M+H]. ^1^H NMR (DMSO-d_6_; 300 MHz): 8.01 (1H, s); 5.00 (1H, m); 4.70 (1H, m); 4.11 (2H, s overlap); 3.77 (1H, s); 3.65 (2H, d)

**Synthesis of 7 oxo CA (16)**

Compound 9 was selectively oxidized according to a literature procedure [7]. A white-yellowish solid was obtained (100 %) and was used without further purification.

**Synthesis of 7 oxo CAME (17)**

Compound 16 was esterified based on a method described in literature [8]. Compound 16 (3 g, 7.379 mmol) was dissolved in a mixture of 0.75 mL 37 % HCl, 7.5 mL dimethoxypropane and 15 mL MeOH. The reaction mixture was stirred for 24h at room temperature, after which the solvents were evaporated and the residue subjected to silica gel column chromatography to afford a white solid (100%). MS: 438.3241 [M+NH4]. ^1^H NMR (DMSO-d_6_; 300 MHz): 4.50 (1H, m); 4.30 (1H, m); 3.55 (3H, s)

**Synthesis of 7 oxo 3α, 12α DiAc CAME (18)**

Compound 17 was subjected to procedure A, yielding a white solid (65.6 %). MS: 522.3414 [M+NH4]. ^1^H NMR (DMSO-d_6_; 300 MHz): 4.95 (1H, m); 4.55 (1H, m); 3.55 (3H, s) 2.02 (3H, s); 1.94 (3H, s)

**Synthesis of 7α OH 3α, 12α DiAc CAME (19)**

Compound 18 was reduced based on a method described in literature [9]. Compound 18 (200 mg; 0.3961 mmol) and NaBH_4_ (28 mg; 0.740 mmol) were dissolved in 5 mL 20% THF in methanol. The reaction mixture was stirred for 4 hours at room temperature under inert atmosphere. Then, the solvents were evaporated and the residue redissolved in 100 mL EtOAc. The organic phase was washed with 100 mL NaHCO_3_, 100 mL brine and 100 mL water. It was then dried over MgSO_4_, concentrated *in vacuo* and subjected to column chromatography to yield a white solid (49.8%). MS: 524.3609 [M+NH4]. ^1^H NMR (DMSO-d_6_; 300 MHz): 4.93 (1H, m); 4.41 (1H, m); 4.25 (1H, m); 3.63 (1H, s) 3.55 (3H, s); 2.02 (3H, s); 1.94 (3H, s)

**Synthesis of 7α Ms 3α, 12α DiAc CAME (20)**

Compound 19 was subjected to procedure C, yielding a white solid (84.3%). MS: 602.3384 [M+NH4]. ^1^H NMR (DMSO-d_6_; 300 MHz): 4.95 (1H, m); 4.75 (1H, m); 4.48 (1H, m); 3.55 (3H, s); 3.14 (3H, s); 2.04 (3H, s); 1.97 (3H, s)

**Synthesis of 7β F 3α, 12α DiAc CAME (21)**

Compound 19 was subjected to procedure D, but no fluorinated reaction product was formed. MS- and NMR-analysis showed formation of elimination side product.

**Synthesis of 7β FCA (22)**

Compound 22 could not be synthesized out of compound 21.

**Synthesis of 3α,7α DiAc 12α OH CAME (23)**

The synthesis of compound 23 was based on a literature procedure [10]. To compound 1 (1 g, 2.366 mmol), 1.25 mL pyridine, 5 mL toluene and Ac_2_O (1.25 mL, 13.2 mmol) was added. The reaction mixture was stirred overnight at room temperature. Then 40 mL of toluene and 50 mL water was added. The organic phase was washed with 50 mL 6 M HCl, 50 mL brine and 50 mL water, after which it was dried with CaCl_2_ and evaporated. The residue was subjected to silica gel column chromatography to afford a white solid (58.2 %). MS: 551.3215 [M+FA-H]. ^1^H NMR (DMSO-d_6_; 300 MHz): 4.73 (1H, m); 4.46 (1H, m); 4.25 (1H, m); 3.77 (1H, m); 3.55 (3H, s); 1.96 (6H, s; overlap)

**Synthesis of 3α,7α DiAc 12α Ms CAME (24)**

Compound 23 was subjected to procedure C, yielding a white solid (62.0%) MS: 602.3403 [M+NH4]. ^1^H NMR (DMSO-d_6_; 300 MHz): 4.96 (1H, m); 4.77 (1H, m); 4.43 (1H, m); 3.55 (3H, s); 3.18 (3H, s); 1.99 (3H, s); 1.94 (3H, s)

**Synthesis of 3α,7α DiAc 12β F CAME (25)**

Compound 23 was subjected to procedure D, yielding a white solid (27.3 %). MS: 531.3121 [M+Na]. ^1^H NMR (DMSO-d_6_; 300 MHz): 4.95 (1H, m); 4.77 (1H, m); 4.47 (1H, m); 3.54 (3H, s); 1.97 (3H, s); 1.94 (3H, s)

**Synthesis of 12β FCA (26)**

Compound 25 was subjected to procedure E, yielding a white solid (58.4 %). MS: 409.2743 [M-H]. ^1^H NMR (DMSO-d_6_; 300 MHz): 11.92 (1H, s); 4.93 (1H, m); 4.37 (1H, m); 4.26 (1H, m); 3.75 (1H, s); 3.64 (1H, s)

**Synthesis of CDCAME (28)**

Compound 27 (CDCA) was esterified as described earlier in literature [1]. A white solid (100 %) was obtained. MS: 441.2774 [M+Cl]. ^1^H NMR (DMSO-d_6_; 300 MHz): 4.28 (1H, m); 4.08 (1H, m); 3.61 (2H, s overlap); 3.55 (1H, s)

**Synthesis of 3α, 7α DiAc CDCAME (29)**

Compound 28 was subjected to procedure A, yielding a white solid (97.3 %). MS: 508.3628 [M+NH4]. ^1^H NMR (DMSO-d_6_; 300 MHz): 4.74 (1H, m); 4.45 (1H, m); 3.55 (3H, s); 1.96 (3H, s); 1.94 (3H, s)

**Synthesis of 3α OH 7α Ac CDCAME (30)**

Compound 29 was subjected to procedure B, yielding a white solid (96.7 %). MS: 483.2885 [M+Cl]. ^1^H NMR (DMSO-d_6_; 300 MHz): 4.71 (1H, m); 4.38 (1H, m); 3.55 (3H, s); 1.96 (3H, s)

**Synthesis of 3α Ms 7α Ac CDCAME (31)**

Compound 30 was subjected to procedure C, yielding a white solid (74.3 %). MS: 544.3317 [M+NH4].^1^H NMR (DMSO-d_6_; 300 MHz): 4.75 (1H, m); 4.41 (1H, m); 3.55 (3H, s); 3.13 (3H, s); 1.97 (3H, s)

**Synthesis of 3β F 7α Ac CDCAME (32)**

Compound 30 was subjected to procedure D, yielding a white solid (53.3%). ^1^H NMR (DMSO-d_6_; 300 MHz): 4.88 (1H, m); 4.75 (1H, m); 3.55 (3H, s); 1.97 (3H, s)

**Synthesis of 3β FCDCA (33)**

Compound 32 was subjected to procedure E, yielding a white solid (77.7 %). MS: 393.2913 [M-H]. ^1^H NMR (DMSO-d_6_; 300 MHz): 11.92 (1H, s); 4.83 (m, 1H); 4.20 (1H, m); 3.63 (s, 1H)





Figure 1: synthesis of 3αFCA precursor for radiosynthesis and reference compound. a: 3,5-DNBA, PPh_3_, THF, 50°C, 48h (78.57 %); b: NaOMe, MeOH, THF, RT, 15 min (100 %); c: 4-DMAP, Ac_2_O, pyridine, RT, overnight (53.9 %); d: AcCl, MeOH, 0°C-> RT, 32h (96.8 %); e: MsCl, pyridine, 0°C -> RT (72.0 %); f: DAST, CH_2_Cl_2_, RT, 15 min (62.6 %); (62.6 %) g: KOH, MeOH, reflux, 45 min (92.0 %).





Figure 2: Synthesis of 3βFGCA precursor for radiosynthesis and reference compound. a: glycine methylester, EEDQ, Et3N, EtOAc, reflux, overnight (100 %); b: 4-DMAP, Ac_2_O, pyridine, RT, overnight (70.0 %); c: AcCl, MeOH, 0°C-> RT, 32h (72.8 %); d: MsCl, pyridine, 0°C -> RT (75.5 %); e: DAST, CH_2_Cl_2_, RT, 15 min (63.0 %); f: KOH, MeOH, reflux, 45 min (75.5 %)





Figure 1: Synthesis of 7βFCA precursor for radiosynthesis and reference compound. a: NBS, NaHCO_3_, H_2_O, RT, 24h (100 %); b: HCl, dimethoxypropane, MeOH, RT, 24h (100 %); c: 4-DMAP, Ac_2_O, pyridine, RT, overnight (65.6 %); d: NaBH_4_, THF, MeOH, RT, 4h (49.8 %); e: MsCl, pyridine, 0°C -> RT (84.3 %); f: DAST, CH_2_Cl_2_, RT, 15 min (no product obtained); g: KOH, MeOH, reflux, 45 min (no product obtained)





Figure 2: synthesis of 12βFCA precursor for radiosynthesis and reference compound. a: Ac2O, toluene, pyridine, RT, overnight; b: MsCl, pyridine, 0°C -> RT (62.0 %); c: DAST, CH_2_Cl_2_, RT, 15 min (27.3 %); d: KOH, MeOH, reflux, 45 min (58.4 %)





Figure 3: Synthesis of 3βFCDCA precursor for radiosynthesis and reference compound. a: HCl, MeOH, reflux, overnight (100 %); b: 4-DMAP, Ac_2_O, pyridine, RT, overnight (97.3 %); c: AcCl, MeOH, 0°C-> RT, 32h (96.7 %); d: MsCl, pyridine, 0°C -> RT (74.3 %); e: DAST, CH_2_Cl_2_, RT, 15 min (53.3 %); f: KOH, MeOH, reflux, 45 min (77.7 %)

# References

1. Májer F, Sharma R, Mullins C, Keogh L, Phipps S, Duggan S, et al. New highly toxic bile acids derived from deoxycholic acid, chenodeoxycholic acid and lithocholic acid. Bioorganic Med Chem. 2014;22: 256–268. doi:10.1016/j.bmc.2013.11.029

2. De Lombaerde S, Neyt S, Kersemans K, Verhoeven J, Devisscher L, Van Vlierberghe H, et al. Synthesis, in vitro and in vivo evaluation of 3β-[18F]fluorocholic acid for the detection of drug-induced cholestasis in mice. PLoS One. 2017;12: e0173529. doi:10.1371/journal.pone.0173529

3. Rohacova J, Marin ML, Martínez-Romero A, O’Connor J-E, Gomez-Lechon MJ, Donato MT, et al. Synthesis of new, UV-photoactive dansyl derivatives for flow cytometric studies on bile acid uptake. Org Biomol Chem. 2009;7: 4973–80. doi:10.1039/b912134j

4. Zhao Y, Zhong Z. Oligomeric cholates: Amphiphilic foldamers with nanometer-sized hydrophilic cavities. J Am Chem Soc. 2005;127: 17894–17901. doi:10.1021/ja056151p

5. Gunasekara RW, Zhao Y. Conformationally switchable water-soluble fluorescent bischolate foldamers as membrane-curvature sensors. Langmuir. 2015;31: 3919–3925. doi:10.1021/acs.langmuir.5b00379

6. Tserng K, Hachey DL, Klein PD. An improved procedure for the synthesis of glycine and taurine conjugates of bile acids. J Lipid Res. 1977;18: 404–407.

7. LF F, S R. Selective Oxidation with N-Bromosuccinimide: cholic acid. J Am Chem Soc. 1949;71: 3935–3938.

8. Neyt S, Vliegen M, Verreet B, De Lombaerde S, Braeckman K, Vanhove C, et al. Synthesis, in vitro and in vivo small-animal SPECT evaluation of novel technetium labeled bile acid analogues to study (altered) hepatic transporter function. Nucl Med Biol. Elsevier B.V.; 2016;43: 642–649. doi:10.1016/j.nucmedbio.2016.07.001

9. Iuliano A, Facchetti S, Uccello-Barretta G. Asymmetric induction by the cholestanic moiety on Tropos species: Synthesis and stereochemical characterization of bile acid-based biphenyl phosphites. J Org Chem. 2006;71: 4943–4950. doi:10.1021/jo0606453

10. Ito A, Ishizaka S, Kitamura N. A ratiometric TICT-type dual fluorescent sensor for an amino acid. Phys Chem Chem Phys. 2010;12: 6641–6649. doi:10.1039/b924176k
